# Responding to the AMR threat: data and information needs of stakeholders working in regional and remote Australia

**DOI:** 10.1017/ash.2024.87

**Published:** 2024-05-27

**Authors:** Matthew Barry Eustace, Lisa Hall, Bhavini Patel, Teresa Maria Wozniak

**Affiliations:** 1 University of Queensland School of Public Health, Brisbane, QLD, Australia; 2 Northern Territory Health, Darwin, NT, Australia; 3 Australian e-Health Research Centre (AEHRC) CSIRO, Brisbane, QLD, Australia

## Abstract

Our qualitative analysis of interviews with remote Australian healthcare professionals found that they require reliable, local antimicrobial resistance (AMR) data reflecting the geographical diversity of the population they serve. The optimal use of AMR data must consider challenges within this setting, including high staff turnover, limited diagnostic capacity, and antibiotic shortages.

## Introduction

The spread of antimicrobial resistance (AMR) represents a significant global public health threat with increased rates of resistance being observed in both the hospital and community settings.^1^ The growing burden of AMR has led to increased rates of hospitalization, longer hospital stays, higher treatment complications, and significant economic impact on the health system.^2^ In Australia, the AMR burden is exceedingly high in remote settings where ongoing monitoring and disease surveillance have historically been limited.^3^

Healthcare delivery challenges in remote areas result in significant delays in microbiological diagnostics compared with urban settings. These delays mean many patients may wait up to 5 days or more for directed antimicrobial therapy after the initial diagnostic test, provided it was accessible to begin with.^4^ In rural and remote Australia, workforce constraints mean remote area nurses and Aboriginal health practitioners often take on the role of primary care providers. To support timely access to medicines, these staff are authorized to supply and administer certain medicines providing they follow specified treatment protocols approved by the Northern Territory Chief Health Officer, such as the Central Australian Rural Practitioners Association (CARPA) manuals.^5^ Although, Aboriginal health practitioners are authorized to supply antibiotics, <1% actually do so as part of their practice.^4^ This is in contrast to urban clinics, where general practitioners are the main prescribers of antibiotics.^6^ Additionally, there are no routine antibiograms produced in Australia for primary care, limiting the available data healthcare providers can access to inform empiric antimicrobial prescriptions.

In response to the identified need for regional and remote healthcare practitioners to access local AMR data at the point of care, the HOTspots Surveillance and Response Program was established in 2018. In 2019, following extensive engagement with local clinicians and policy, the HOTspots digital surveillance platform was launched in clinical practice.^7^ AMR surveillance data from HOTspots are provided to the national AMR surveillance system—Antimicrobial Usage and Resistance in Australia program^8^. In response to these positive actions in monitoring and responding to AMR in regional and remote Australia, we aimed to address the knowledge gap and evaluate the data and information needs of clinicians and policymakers working to reduce the AMR threat in these settings.

## Methods

### Design

A qualitative study design was used to assess transcribed semi-structured interviews (n = 13) utilizing interpretive description techniques.

### Study setting and participants

This study was conducted in remote Australia. Interview participants were end users of AMR data and included laboratory/pathology data custodians, healthcare providers responsible for clinical management of AMR patients, policymakers responsible for strategic response to AMR, and public health practitioners responsible for population-level disease control.

### Data collection

Data from transcribed semi-structured interviews conducted in 2019 as part of an evaluation of an AMR surveillance system were reviewed to identify comments that related to data use and information needs of the interview participants.

### Data coding and analysis

Interviews were recorded and transcribed verbatim. Interview transcripts were reviewed independently by 2 authors (M.E and T.W) who extracted preliminary themes. A process of interpretive description, a method of intellectual inquiry whereby researchers constantly question and reconsider their findings, was then undertaken with 3 authors (M.E, T.W, and L.H). The independently coded data was critically analyzed using this framework until a consensus on themes was reached.

### Ethical considerations

This study received ethical approval from the Human Research Ethics Committee of the NT Department of Health and Menzies School of Health Research (approval number 2019-3425).

## Results

13 semi-structured, individual and group interviews lasting between 8 and 45 minutes were conducted with a total of 18 participants. Our analysis identified 5 key themes: (1) data needs, (2) data governance and management, (3) data confidence, (4) ability to act on evidence including resourcing, and (5) technology challenges (Table 1).

**Table 1.** Identified themes and challenges of clinicians and policymakers to support action on AMR in remote Australia

Interview excerpts	Challenges identified
**Theme 1—Data needs**	
“So, [current surveillance platform] doesn’t get [data from] any of our hub labs…. So, if we do get an MRSA from community, it doesn’t go to [a surveillance platform], not even the data, nothing.” *(pathology data custodian)* “…understanding what’s happening in various health districts or various regions across [remote Australia] at a population level will be really helpful…” *(public health responder)* “…[in] hospital if there are clusters of resistant organisms… that’s flagged with infection control unit and then they’ll institute… outbreak measures, they’ll investigate, and they’ll do a whole lot of different measures to try and contain that, and look for other potential sources of infection… But I don’t believe it actually goes anywhere beyond the hospital.” *(healthcare provider)*	Lack of community-level AMR data Underrepresentation of AMR data from remote Australia at national-level decision-making
**Theme 2—Data governance and management**	
“I have been muddling through trying to work out some of the logistics because a lot of [community] samples go to a lab service in another state and then mostly hospital samples to our jurisdictional pathology service.” *(public health responder)* “…if it comes from a remote town to a regional centre… it’s three days by the time it gets here, then it’s 48 hours before it’s finalized. That’s five days that the patient hasn’t had [directed] treatment.” *(pathology data custodian)*	Jurisdictional challenges Cost Role of regulation, standardization, and interoperability
**Theme 3—Data confidence**	
“a confidence level … would give clinicians more of a sense of what to do in a situation… [with] new technologies in healthcare, clinicians want to understand what’s going on behind the data and recommendations.” *(healthcare provider)* “…if you’re in a remote area, you’re going to follow CARPA. If you’re in an urban area and treating an appropriate population, you’ll probably use therapeutic guidelines.” *(healthcare provider)*	Reliability and validity of data Data transparency Engagement with key stakeholders
**Theme 4—Ability to act on evidence, including resourcing**	
“AHPs and RANs are fantastic at doing something that doctors are bad at, which is following protocols and minimising variations in care.” *(AMR policymaker)* “Sometimes it’s also just a case of what you’ve physically got in stock. Again, with the delays in ordering stuff in [and shortages of key antibiotics]. … if you’re just out of something on a particular day … you’ve got to come up with an alternative appropriate choice. So certainly, remote that definitely makes a difference because … there’s no alternative places, if you don’t have it in the pharmacy room that day, then yeah, they don’t have it.” *(healthcare provider)* “…we don’t have access to a lot of the stock that requires stewardship in the hospitals… We were coming up with a plan recently for a patient who was at high risk of developing wound infections and determining what sort of antibiotics we might put him on. And that was really done in conjunction with also looking at the physical shelf and seeing what we had.” *(healthcare provider)*	Workforce shortages/high staff turnover Scope of practice Limited remote resources
**Theme 5—Technology challenges**	
“…for the general population General Practitioners, there isn’t utility at the moment [for a novel AMR platform] and that’s partly because it’s another system and other thing you have to log into, there’s quite a learning curve to be able to get information out that’s useful.” *(healthcare provider)* “[collection and provision of AMR data] I think genuinely has benefits in terms of patient outcomes and not just immediately for the patient in front of you, but also on a population health level, the challenge is making that useable.” *(healthcare provider)*	Cognitive overload Login fatigue Interface and connectivity challenges

Note. AMR, antimicrobial resistance; CARPA, Central Australian Rural Practitioners Association; MRSA, methicillin-resistant *Staphylococcus* aureus; AHP, Aboriginal health practitioner; RAN; Remote Area Nurse.

## Discussion

In remote Australia, clinicians and policymakers face the challenge of supporting a patient population with a disproportionately high burden of chronic disease and infectious diseases and an exceedingly high burden of AMR.^9^ This study highlights the need for reliable, local, and geographically representative data to guide effective clinical and policy decision-making in the face of the unique challenges of regional and remote Australia. We identified 3 crucial challenges—limited workforce, delayed confirmation of causative pathogen, and frequent shortages of antibiotics.

Remote Australia faces significant challenges due to a limited workforce and high staff turnover.^10,11^ In these regions, the primary healthcare workforce consists mainly of remote area nurses and Aboriginal health practitioners, with support from visiting medical staff. These healthcare professionals follow local treatment guidelines such as the CARPA manual. Timely access to local AMR data to inform clinical practice is crucial and is most practical when integrated into local treatment guidelines and clinical pathways, such as the Primary Health Network Health Pathways.

In remote areas of Australia, the distance between community clinics and diagnostic laboratories often leads to significant delays in receiving antimicrobial susceptibility test results. This means clinicians must make the difficult choice of an antibiotic at the point of care prior to receiving the antimicrobial susceptibility results. To support their clinical judgment, it is important to provide accessible data on the region’s AMR patterns. This helps ensure the optimal choice of antibiotics when local diagnostic capabilities are limited.

Lastly, we identified that healthcare professionals in regional and remote Australia often struggle to maintain a steady supply of medications including antibiotics. Due to geopolitical unrest and disrupted supply chains, shortages of critical antibiotics have become more common in Australia.^12^ Participants in this study noted issues such as supply shortages, challenges with stockpiling, and fluctuations in stock rotation costs in their clinics and local pharmacies. Providing policymakers and those in charge of medicines management with region-specific AMR data can help them assess the need for specific antibiotics to treat their local patient population. This would aid early preparation and facilitate seeking alternative antibiotic supply chains in the event of potential shortages.

This study is limited to the views of participants which may not be representative of all the views of healthcare professionals within regional and remote Australia. There was an unequal representation of health policymakers in our study, and only 1 was included in the analysis. However, the study had a good representation of clinical participants, who face significant challenges in responding to AMR in regional and remote Australia.

## Conclusion

This study is the first to examine the AMR data and information needs of healthcare professionals working in regional and remote Australia. Understanding healthcare professionals’ perspective of what data and information needs they have will ensure that local surveillance systems such as HOTspots meet the needs of the end users. These findings will guide the integration of AMR surveillance data into clinical practice, prescribing guidelines, and policy decisions to strengthen local and national response to the AMR threat.
